# Does having a cat in your house increase your risk of catching COVID-19?

**DOI:** 10.1016/j.onehlt.2022.100381

**Published:** 2022-03-25

**Authors:** Valerie Allendorf, Nicolai Denzin, Franz J. Conraths, Lisa A. Boden, François Elvinger, Ioannis Magouras, Arjan Stegeman, James L.N. Wood, Ana Carvajal Urueña, Katherine E.F. Grace, Katharina D.C. Stärk

**Affiliations:** a Friedrich-Loeffler-Institut, Institute of Epidemiology, Greifswald – Insel Riems, Germany; b Friedrich-Loeffler-Institut, Institute of International Animal Health & One Health, Greifswald – Insel Riems, Germany; c Global Academy of Agriculture and Food Security, University of Edinburgh, Edinburgh, UK; d Cornell University, Department of Population Medicine and Diagnostic Science, Ithaca, USA; e City University of Hong Kong, Department of Infectious Diseases and Public Health, Hong Kong Special Administrative Region; f Universiteit Utrecht, Department of Population Health Sciences, Utrecht, the Netherlands; g Disease Dynamics Unit, University of Cambridge, Department of Veterinary Medicine, Cambridge, UK; h Universidad de León, Department of Animal Health, León, Spain; i Epidemiology and Risk Policy Advice, APHA, Westminster, UK; j Federal Food Safety and Veterinary Office (BLV), Department of Animal Health, Bern, Switzerland

**Keywords:** SARS-CoV-2, One health, Cat, Risk assessment, Delphi, Zoonosis

## Abstract

Due to the zoonotic origin of SARS-Coronavirus 2 (SARS-CoV-2), the potential for its transmission from humans back to animals and the possibility that it might establish ongoing infection pathways in other animal species has been discussed. Cats are highly susceptible to SARS-CoV-2 and were shown experimentally to transmit the virus to other cats. Infection of cats has been widely reported. Domestic cats in COVID-19-positive households could therefore be a part of a human to animal to human transmission pathway. Here, we report the results of a qualitative risk assessment focusing on the potential of cat to human transmission in such settings. The assessment was based on evidence available by October 2021.

After the introduction of SARS-CoV-2 to a household by a human, cats may become infected and infected cats may pose an additional infection risk for other members of the household. In order to assess this additional risk qualitatively, expert opinion was elicited within the framework of a modified Delphi procedure. The conclusion was that the additional risk of infection of an additional person in a household associated with keeping a domestic cat is very low to negligible, depending on the intensity of cat-to-human interactions. The separation of cats from humans suffering from SARS-CoV-2 infection should contribute to preventing further transmission.

## 1 Introduction

The emergence of the novel severe acute respiratory syndrome coronavirus 2 (SARS-CoV-2) in 2019, most probably from spill-over infection from an as yet unknown animal host into the human population [1,2] raised the question of the role that domestic animals might play in the course of the pandemic [3,4]. Experimental infections of a range of animal species revealed high susceptibility of the domestic cat (*Felis catus*) and also demonstrated between cat transmission of the virus [[5], [6], [7], [8], [9]]. Concomitantly, these findings are complemented by a growing number of case reports on natural infections of cats [[10], [11], [12], [13]] and by surveys on the seroprevalence of SARS-CoV-2 in cats living in infected households [[14], [15], [16]]. In 2019, 23.5% (median, range 11% (Spain) - 47% (Romania)) of the 195.4 million households in the European Union owned at least one cat [17]. This large proportion of households with domestic cats emphasizes the urgent need for One Health investigations to assess and communicate the risk of cat to human transmission [18].

The secondary attack rate (SAR) of SARS-CoV-2 within an average European household after the introduction of the virus with an index case has been investigated in numerous studies [[19], [20], [21]]. The average SAR was 16.6% (95% CI, 14.0%–19.3%), but it differed widely between various age groups and was influenced by relationship patterns, reaching 37.8% (95% CI, 25.8%–50.5%) in partnerships [22]. Additionally, the infecting viral variant was shown to have an impact on the SAR [23]. The presence of clinical signs increases the probability of transmission [21,24]. Similarly to human to human transmission, it can be assumed that transmission to cats may occur via droplets, aerosol or fomites [25]. Although it has not been possible to identify a single main transmission route, there is evidence that the probability of infecting a cat increases with contact intensity and duration [26,15]. Several case reports describe mild to moderate signs in the infected cats, including respiratory [27,28], gastro-intestinal [29,30] or general non-specific [31,32] signs of disease. Severe clinical signs and lesions similar to those described in severe human COVID-19 cases have been described in a household cat with hypertrophic cardiomyopathy, with high viral loads in multiple tissues and potential for elevated viral shedding [33], although an asymptomatic course of infection has been reported in the majority of cases [34]. In natural infections, viral RNA loads measured by RT-qPCR in different samples, mainly of the upper respiratory tract or the rectum, reached up to 10^8^ RNA copies/ml [13] or 10^3^ RNA copies/ml, respectively [10,7] in the acute phase of viral replication, which was shown to last for 3–6 days [6,8]. In this stage of shedding, cats may transmit the virus to co-housed cats [35,8]. Experimental passaging of the virus in cats was shown to attenuate further transmission already from the second passage onwards, without any detectable changes in the viral RNA sequence [5]. Spillback transmission from cats to humans has not been reported so far. Nonetheless, reports of human infections deriving from infected minks on a fur farm [36], hamsters sold in a pet shop [37], and most recently wild white-tailed deer [38] demonstrate the potential risk animals might pose in onward transmission chains and possible variant alteration.

This study aimed to assess the added risk of infection of household members resulting from the presence of domestic cats after introduction of COVID-19 to the household via a human index case. This risk assessment is intended to provide policy makers and veterinarians with guidance on appropriate risk mitigation measures for cat owners. There are numerous gaps of evidence or knowledge regarding aspects of the risk pathway, deriving from the limited time and ability to perform large-scale observational studies on frequency and mode of interspecies transmissions. Accordingly, the assessment was performed qualitatively in the framework of a formal and structured elicitation of expert opinion [39]. In decision theory, probability judgements by experts are seen as the result of heuristics. Expert judgment may be calibrated by either comparison with a similar event [40] or by the construction of mental models under different initial conditions and operating parameters [41]. A modified Delphi approach, which is frequently used in human health risk analysis [42,43], systematically captures and merges the judgements of several experts while attempting to exclude bias resulting from group discussions. Through aggregation of independently provided opinions of different experts on disassociated single steps within a complex scenario, the combined probability estimate for the overall risk in question is supposedly of the highest possible accuracy in data-scarce environments [44].

The objective of this study was to qualitatively assess the additional risk the presence of a cat in a household poses with respect to onward transmission of a SARS-CoV-2 infection.

## 2 Materials and methods

A qualitative risk assessment was conducted by a group of Veterinary Public Health (VPH) specialists, a convenience sample of individuals who volunteered after a call for interest issued by the European College of Veterinary Public Health (ECVPH, https://ecvph.org/) among its members. The group met regularly over the course of a seven-month period starting in February 2021. The ten specialists were affiliated with universities, research institutes or governmental institutions for VPH in six different countries on three continents. All participants were dealing actively with the SARS-CoV-2 pandemic as part of their jobs, be it in a risk assessment or risk management context. As quantitative data on human to cat transmission and vice versa were sparse or non-existent, a qualitative approach for risk assessment was chosen. An elicitation of expert opinion was performed within a framework of a modified Delphi procedure [42,45].

**Fig. 1:**
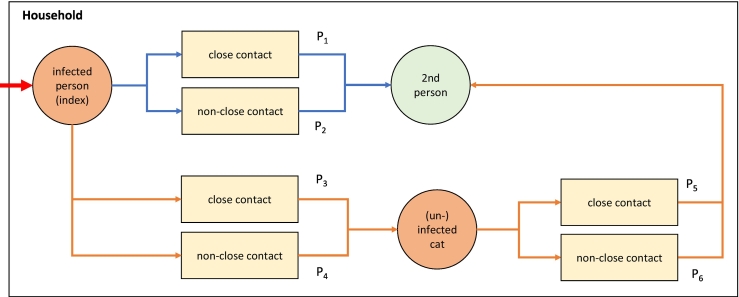
Scenario tree model on the risk pathways of inter-human transmission (blue) and the additional pathways to be considered in the presence of a domestic cat (orange). (For interpretation of the references to colour in this figure legend, the reader is referred to the web version of this article.)

All meetings were held online, using video conferencing software. Consensus on the scope and objective of the study were achieved in the initial meeting. It was agreed to carry out a risk analysis orientating towards the guidelines of the World Organization for Animal Health (OIE) [46]. The risk question was stated as: “What is the additional risk of a zoonotic transmission to a human in a household with a human index case of SARS- CoV-2 infection if a domestic cat is present?” The hazard was defined as SARS-CoV-2. For the sake of a robust, straightforward approach, an average transmissibility with respect to the variants of SARS-CoV-2 was assumed. Over the next meetings, a scenario tree was drafted covering the main pathways of risk (Fig. 1). Bearing in mind the limited data availability, the pathways were subsequently broken down into the relevant steps or events leading to the outcome described in the risk question. To each step, a probability P_n_ was designated, to be estimated throughout the process of expert elicitation. Publications on the probability of transmission at the different steps in the pathways informed the assessment.

The definitions of the general prerequisites and categories applied within the investigated scenario were agreed upon. A household was assumed to consist of at least two persons living in a dwelling, sharing at least kitchen, bathroom and a common living space, and observing the general rules of personal and household hygiene. Any domestic cat belonging to the household was assumed to be fed by its owner, to be friendly towards the household members, spending time sleeping or resting within the household and using the cat litter box. The broad spectrum of possible human-cat-interactions was broken down into two risk behaviour categories, according to the intensity of the human-cat-interface. It was discriminated between a close-contact category, including all interactions taking place within a range of 1 m around the two interacting subjects, and a non-close contact category, summarizing the interactions at more than 1 m distance and the non-simultaneous use of rooms or household equipment. In close-contact situations, it was assumed that the three considered transmission pathways, i.e. droplets, aerosols and fomites, were possible. In non-close contact situations, the possible transmission pathways were reduced to airborne or fomite transmissions. The index case was defined as the first case of COVID-19 occurring in the household, introducing the hazard into the setting. An average course of disease of this index case was assumed, without initial knowledge of infection of this index person.

When the scope of the risk scenario had been finalized and agreed upon, each expert was asked to estimate the disassociated probabilities P_n_ of the individual steps in the risk pathways, applying the OIE scale of qualitative risk descriptors. All experts performed the assessment independent of each other. OIE standards [47] were used as qualitative descriptors for the estimates (Table 1). For each of their estimates, the experts were asked to state the main reasons, why they came to their conclusion. An excel sheet [48] was emailed to each expert, which they were asked to fill in and return within four weeks. After week three, a reminder was sent.

**Table 1:** Qualitative descriptors of probabilities according to OIE standards [47].

Descriptor	OIE definitions
Very high	The event occurs almost certainly.
High	The event occurs very often.
Moderate	The event occurs regularly.
Low	The event is rare but does occur.
Very low	The event is rare but cannot be excluded.
Negligible	The event is so rare that it does not merit to be considered.

After completion of the first round of expert opinion elicitation, all estimates were aggregated in an anonymous form and presented to the expert panel in the following online meeting. Subsequently, experts were asked to revise their opinion in the light of the panel's first vote, and to submit a final estimate for each individual probability, accompanied again by the main reasons for the respective decision.

An aggregation of an expert panels' estimates of individual probabilities may be performed mathematically or behaviourally [49]. The conducted protocol combines a behavioural step through application of a second round of estimation with a mathematical aggregation in the case of a dissent after the second and final round of estimation. The validity of each probability was evaluated with respect to both, the degree of agreement among the expert opinions and the available scientific evidence. The degree of agreement was calculated with Leik's D as a measure of ordinal dispersion [50], using the agrmt-package [51] in RStudio [52]. As Leik's D takes values between 0 – total agreement – and 1 – total dispersion, it can be interpreted as a percentage value of dispersion. Inversely, by subtracting Leik's D from 1, it can be interpreted as the percentage degree of agreement. These degrees of agreement were then translated into qualitative descriptors according to Table 2, which was discussed and agreed upon.

**Table 2:** Translation of the degree of agreement calculated by using Leik's D into qualitative descriptors of expert agreement.

Agreement	(1-Leik's D)
Low	< 50%
Medium	50–90%
High	> 90%

To describe the uncertainty deriving from scientific evidence, the available literature was evaluated regarding data availability (type of data, amount, quality) and data variability (consistency) (Table 3) based on a scale proposed by the European Food Safety Authority (EFSA) in 2006 [53].

**Table 3:** Uncertainties deriving from availability of published studies, based on the scale proposed by EFSA [53].

Uncertainty	Data availability (type, amount, quality)	Data variability (consistency)
Low	scarce or no data available; evidence not provided in references but rather in unpublished reports, based on observation	authors report similar conclusion
Medium	some but no complete data available, evidence provided in small number of references	authors report conclusions that vary from each other
High	solid and complete data available; strong evidence provided in multiple references	authors report conclusions that vary considerably between them

After the assessment of the individual probabilities, the qualitative estimates of the zoonotic domain of the scenario consisting of two steps and with that, two individual probabilities P_n_ were combined using the structure of a combination matrix (Table 4) as proposed by Gale et al. [54]. This matrix is built on the fact that probabilities range from 0 to 1, which implies that the mathematical product (and thus its qualitative equivalent) of two probabilities may not be higher than the lower probability, and additionally allows for an improved combination when multiplying values of the lower end of the qualitative scale. Applying this matrix, the modes of the assessed probabilities as well as the worst and best case estimates were combined.

**Table 4:** Risk combination matrix according to Gale et al. [54].

Event 2	Negligible	Very low	Low	Moderate	High	Very high
Event 1						
Negligible	negligible	negligible	negligible	negligible	negligible	negligible
Very low	negligible	negligible	negligible	very low	very low	very low
Low	negligible	negligible	very low	low	low	low
Moderate	negligible	very low	low	moderate	moderate	moderate
High	negligible	very low	low	moderate	high	high
Very high	negligible	very low	low	moderate	high	very high

Overall probabilities of the different pathways were then expressed as point estimates with the respective range of worst and best case estimates. The assessed uncertainties regarding evidence and expert agreement were summarized and combined in the matrix (Table 5) proposed by the Intergovernmental Panel on Climate Change (IPCC) [55]. The results derived from the elicitation of expert opinion were then reported together with the confidence in the overall assessment.

**Table 5:** Matrix for combining uncertainties deriving from available evidence and degree of expert agreement according to the IPCC [55].

Evidence →	Limited	Medium	Robust
Agreement ↓			
Low	Low	Low	Medium
Medium	Low	Medium	Medium
High	Medium	Medium	High

## 3 Results

### 3.1 Definition of close-contact scenario (droplet, aerosol or fomite transmission)

Experts considered the following interactions as possible risks for transmission in both directions: scent marking (cat face to human face), lifting the cat, having it on one's lap, stroking or cuddling; sharing of bed or seating furniture simultaneously. Furthermore, for human-to-cat transmission, letting the cat eat leftovers from the owner's plate was also considered. Allowing the cat on the dining table and stroking the cat's coat after it grooms itself were considered risk factors in the cat-to-human transmission pathway.

### 3.2 Definition of non-close contact scenario (aerosol or fomite transmission)

Experts considered the cat moving mostly on the floor, sharing enclosed spaces simultaneously and non-simultaneously with people, petting the cat, feeding the cat, and specifically for the cat-to-human direction of transmission, cleaning the cat's litter box. Fomite transmission risk was considered to be associated with the size of the living space, degree of cleanliness and the degree of personal hygiene (washing hands) within the assessed household.

### 3.3 Elicitation of probability estimates

Of the ten experts in the panel, nine submitted the completely filled-out questionnaire in both given time periods for the first and second round of opinions.

**Fig. 2:**
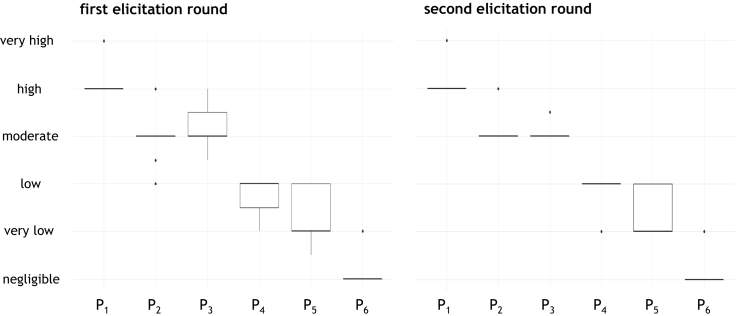
Boxplots of the results of the first (left) and second round of expert opinion elicitation.

In the first round of expert elicitation, there was some disagreement between experts in their estimates, although difference on the ordinal scale of risk were limited. In the second round, six experts confirmed their initial estimates, while three decided to change their opinion concerning one or more probabilities. In two cases, the estimates of the probability that SARS-CoV-2 is transmitted from the index person to a second household member under distance conditions (P_2_) and the estimates of the probability that SARS-CoV-2 is transmitted from the index person to a domestic cat under distance conditions (P_4_) were increased from low to moderate respectively from very low to low. In one case, the estimate of the probability that SARS-CoV-2 is transmitted from a domestic cat to a second household member under close-contact-conditions (P_5_) was increased from negligible to very low. One expert decreased the assessment of the probability that SARS-CoV-2 is transmitted from the index person to a domestic cat under close-contact-conditions (P_3_) from high to moderate. This led to a reduction of variance of the respective group estimates (Fig. 2).

### 3.4 Aggregation of estimates

The final estimates of the experts were aggregated and analysed concerning the underlying uncertainties as described above. The results are provided in Table 6.

**Table 6:** Final estimates and related uncertainties assigned to the steps of the risk pathways.

		Probability	Mode	„Worst case“ estimate	„Best case“ estimate	Availability data	Variability data	Agreement experts	Confidence
Human to human	P_1_	… that SARS-CoV-2 is transmitted from index person to 2nd household member under **close-contact-conditions**	***high***	*very high*	*high*	*high*	*low*	*high*	*high*
	P_2_	… that SARS-CoV-2 is transmitted from index person to 2nd household member under **distance conditions**	***moderate***	*high*	*moderate*	*high*	*low*	*high*	*high*
Human to cat	P_3_	… that SARS-CoV-2 is transmitted from index person to domestic cat under **close-contact-conditions**	***moderate***	*high*	*moderate*	*medium*	*medium*	*high*	*medium*
	P_4_	… that SARS-CoV-2 is transmitted from index person to domestic cat under **distance conditions**	***low***	*low*	*very low*	*low*	*low*	*high*	*medium*
Cat to human	P_5_	… that SARS-CoV-2 is transmitted from domestic cat to 2nd household member under **close-contact-conditions**	***very low***	*low*	*very low*	*low*	*NA*	*medium*	*low*
	P_6_	… that SARS-CoV-2 is transmitted from domestic cat to 2nd household member under **distance conditions**	***negligible***	*very low*	*negligible*	*low*	*NA*	*high*	*medium*

### 3.5 Overall risk estimates of the qualitative assessment

Concerning the standard domain of direct human-to-human transmission, the transmission risk (SAR) was assessed to be high (range: high - very high) under close-contact (proximal) conditions and moderate (range: moderate – high) under non-close contact (distal) conditions. The confidence in the risk assessment of this domain was high.

The additional risk posed by a cat in a household and thus answering the risk question is derived from the zoonotic domain of the scenario. Within the zoonotic domain, the combination of the probabilities assigned to the two steps involved (P_3_ / P_4_ multiplied with P_5_ / P_6_) yielded the following estimates of the additional risk of infection of a second person in the household:

→ very low (range: very low – low), if both household members have close contact with the cat (P_3_*P_5_),
→ negligible (range: negligible - very low), if only the index case keeps physical distance from the cat (P_3_*P_6_),
→ negligible (range: negligible - very low), if only the second household member keeps physical distance from the cat (P_4_*P_5_) and
→ negligible, if both household members keep physical distance from the cat (P_4_*P_6_).

The confidence in these assessments is considered as medium (to low).

## 4 Discussion

The approach undertaken in this study yielded a qualitative assessment of the additional risk the presence of a cat in a household poses with respect to the SAR of a SARS-CoV-2 infection. Depending on the assumed intensity of interaction between the involved persons and the cat, the additional risk was estimated as very low in a setting in which household members have close contact to the cat whereas it was estimated negligible in settings in which household members keep physical distance to their cat.

The probabilities estimated in this risk assessment should be understood as additional risks, contrasted against the unavoidable baseline risk of intra-species, i.e. human-to-human transmission in a standard household. The latter risk was estimated as high to very high in a close contact scenario. In the light of this baseline risk, the additional risk associated with the presence of a cat in the household is estimated to be far smaller. The risk posed by a cat will be further reduced by complying with standard recommendations concerning the interaction with animals in the context of SARS-CoV-2 (e.g. recommendations issued by the OIE [56]). Separation from the index patient should apply to all household members, including the cat, and an enhanced attention to hygiene and disinfection within the household should be beneficial for prevention of fomite transmission. Furthermore, if possible, the cat should be kept indoors for the period of household quarantine to prevent spread to cats outside of the household.

Due to the lack of data, especially concerning the characteristics of SARS-CoV-2 related to the host domestic cat, some simplifications and generalizations had to be made in the analysis, and knowledge gaps had to be bridged by expert opinion. Accordingly, the reported assessment is limited by the uncertainties as described. Nevertheless, considering the overall high degree of agreement between experts, the confidence in this assessment is medium. The estimates of the probability that SARS-CoV-2 is transmitted from a domestic cat to a second household member under close-contact-conditions (P_5_) showed a high degree of uncertainty, with consequent low confidence in the assessed probability, reflecting the urgent need for observational studies focusing on this interface.

There is limited information on the influence of SARS-CoV-2 characteristics, and in particular the influence of constantly emerging variants, on transmission and infection probabilities in cats. As with the case of farmed mink [36] or pet hamsters [37], circulation of the virus in a larger cat population could persist over time, followed by zoonotic transmission to humans with unknown consequences. A mink-derived virus variant was shown to lead to enhanced replication and higher morbidity in ferrets, whereas it was attenuated for replication in human airway epithelial cells [57]. Nonetheless, this scenario seems unlikely, as it was shown that with serial passaging of the virus in cats, transmissibility and pathogenicity were significantly reduced after the second passage [5]. Moreover, cats are rarely kept under conditions similar to the high density and low hygiene conditions reported from mink farms [36] or hamster breeding facilities [37].

The probability of onward transmission from human to human within a household was shown to be associated with the severity of symptoms [22], but the contributions of asymptomatic infections to community transmission are difficult to estimate. Asymptomatic and pre-symptomatic patients regularly transmit SARS-CoV-2 efficiently to others [58]. For this overall heterogeneity of clinical manifestations with unknown influence on SAR, an average index patient was assumed. It was not differentiated between different age groups of the index case, although this might have an effect on the course of disease and its severity on the one hand, and the type and intensity of risk-related interactions with the cat on the other hand. For the probability of transmission from an average index case to a cat, the kind of contact behaviour, its intensity and frequency were summarized into two main risk categories.

Intrinsic characteristics of the cat, like breed, age, medical preconditions, etc. were excluded from the risk assessment to ensure a parsimonious model. However, there is also high uncertainty about the impact of these characteristics on susceptibility to the virus, making it difficult to meaningfully include them in the risk pathway. In the case of an infected cat, a subclinical to mild clinical disease was assumed based on the data reported so far [9], although severe disease has been described [33]. In experimental settings, cats showed no clinical symptoms [5,6]. On the contrary, detection of feline SARS-CoV-2 infections in the field by veterinary practitioners is often preceded by suspicious symptoms, triggering laboratory diagnosis. These confirmed cases probably constitute only the tip of the iceberg, as asymptomatically infected cats are not presented to veterinary practitioners. More active surveillance studies in COVID-19 affected households investigating the transmission processes at the human-animal interfaces are necessary to overcome the reporting bias of passive surveillance systems and to determine the prevalence of SARS-CoV-2 infections in exposed cats. Experts agreed that the risk of zoonotic transmission from a cat to a second, previously uninfected person in the household, was negligible to very low depending on the intensity of contact (see Table 6). These estimations were lower than for the zoonotic human-to-cat transmission, mainly because cats have only 10% of the respiratory minute volume of a human, although high viral loads in expired air may occur. The probability of aerosol transmission from a cat to a person is therefore probably of much less importance as compared to a human-to-human transmission scenario. Nonetheless, droplets expelled due to mild respiratory symptoms, grooming of fur with potentially infectious saliva and excretion of potentially infectious faeces were identified as potential sources of infection for immunologically naïve humans, even without direct and very close (face-to-face) contact.

It is imperative to note that the successful transmission of the virus to the cat by the index case is the basic condition on any further considerations within the zoonotic domain of this risk assessment. Being assessed as “moderate” in the close-contact scenario, this might be the most relevant of the assessed probabilities, and therefore the one which should be paid attention to the most. The possible relative contribution of this transmission pathway might even increase if the second person in the household is keeping distance from the patient, but at the same time also has close contact with the cat, maybe even intensified due to alienation from the human co-habitant, with the cat then serving as a potential vector. By efficiently preventing a close contact of the cat to the index patient, the infection risk can be reduced considerably. The overall risk of inter-species transmission especially concerning fomite transmission can be further reduced by following general rules of infection prevention, such as washing hands after cleaning the litter box and physical contact with the cat.

Although expert opinion elicitation is frequently applied in risk assessments in the absence of specific information, there are limitations to this approach. The selection of competent experts is crucial. The group of experts involved in this study self-selected to participate in the elicitation. Since all members of the group are specialists in the field of epidemiology and are involved in research related to SARS-CoV-2 it may be assumed that the group consisted of competent members. The group developed a precise outline of the risk pathways and extensive definitions of the steps the experts had to assess. When it came to the estimation of probabilities itself, the influence of opinion leaders on individual opinions, which is a common phenomenon in group assessments, was minimized through the Delphi process. In both rounds of opinion elicitation, the group members were asked to provide their estimates individually and independently and for the second round, only summary statistics of the first round were provided for their orientation. The second round of opinion elicitation improved the degree of consensus to some extent. While differing quantitative estimates of a group routinely are aggregated by calculating arithmetic means and standard deviations, and may thus be expressed as probability distributions [45], qualitative approaches lack a standard procedure for dealing with dissents. However, dissents may reflect the uncertainty in a data-sparse environment. Accordingly, the majority vote was chosen as the aggregated point estimate for the different probability estimates P_n_, and the respective best-case and worst-case estimates as descriptors of the range of opinions. Due to the lack of evidence in the zoonotic domain of the scenario, the confidence in the risk assessment did not exceed the medium range of the scale, whereas the confidence in the estimates of the human domain, which is more intensely studied, was much higher.

The presented qualitative assessment may be further developed and translated into a quantitative approach, as soon as more empirical data on the occurrence and probability of human-to-cat and cat-to-human transmission become available. During the study period, quantitative results from specific studies conducted in various countries continually emerged. These were integrated as they became available, and uncertainties are expected to be reduced continuously over the coming months.

## 5 Conclusion

According to the presented assessment, domestic cats as additional source of infection for immunological naïve household members play a very low to negligible role. Nonetheless, a zoonotic transmission is plausible under unfavourable circumstances, but there is currently no reported evidence to support this. The presence of clinical disease in the infected cat may contribute to an increase of the transmission risk to the second household member. In addition, the spectrum of interactions between cats and their owners is broad, and the distribution of certain interaction patterns is so far not well described. A cat-human-relationship of extraordinary high degree of closeness as reported by Chomel et al. [59], may lead to an increase in transmission risk in both directions. Especially, if the infection of the index case is known or confirmed, transmission probabilities may be reduced considerably, if established general rules of hygiene are consistently applied. Recommendations concerning the isolation of infected humans, general hygiene rules when interacting with pets and regarding potentially infected animals should be followed.

## Funding

This work was funded by the German Federal Ministry of Education and Research within the COVMon Project, being part of the InfectControl2020 Initiative (BMBF grant no. 03COV16D).

## Author statement

We the undersigned declare that this manuscript is original, has not been published before and is not currently being considered for publication elsewhere.

We confirm that the manuscript has been read and approved by all named authors and that there are no other persons who satisfied the criteria for authorship but are not listed. We further confirm that the order of authors listed in the manuscript has been approved by all of us.

We understand that the Corresponding Author Valerie Allendorf is the sole contact for the Editorial process. She is responsible for communicating with the other authors about progress, submissions of revisions and final approval of proofs.

## Declaration of Competing Interest

The authors declare no conflicts of interest.
